# Conventional Machine Learning versus Deep Learning for Magnification Dependent Histopathological Breast Cancer Image Classification: A Comparative Study with Visual Explanation

**DOI:** 10.3390/diagnostics11030528

**Published:** 2021-03-16

**Authors:** Said Boumaraf, Xiabi Liu, Yuchai Wan, Zhongshu Zheng, Chokri Ferkous, Xiaohong Ma, Zhuo Li, Dalal Bardou

**Affiliations:** 1 Beijing Lab of Intelligent Information Technology, School of Computer Science, Beijing Institute of Technology, Beijing 100081, China; said.boumaraf@bit.edu.cn (S.B.); liuxiabi@bit.edu.cn (X.L.); zhengzhongshu@bit.edu.cn (Z.Z.); 2 Beijing Key Laboratory of Big Data Technology for Food Safety, Beijing Technology and Business University, Beijing 100048, China; 3 Laboratoire des Sciences et Technologies de l’Information et de la Communication LabSTIC, Université 8 Mai 1945 Guelma, BP 401, Guelma 24000, Algeria; ferkous.chokri@univ-guelma.dz; 4 Department of Imaging Diagnosis, National Cancer Center/National Clinical Research Center for Cancer/Cancer Hospital, Chinese Academy of Medical Sciences and Peking Union Medical College, Beijing 100021, China; 5 Department of Pathology, National Cancer Center/National Clinical Research Center for Cancer/Cancer Hospital, Chinese Academy of Medical Sciences and Peking Union Medical College, Beijing 100021, China; lizhuo@cicams.ac.cn; 6 Department of Computer Science and Math, Abbes Laghrour University, Khenchela 40000, Algeria; dalal_bardou@yahoo.fr

**Keywords:** conventional machine learning, deep learning, transfer learning, breast cancer, histopathological images, visual explanation

## Abstract

Breast cancer is a serious threat to women. Many machine learning-based computer-aided diagnosis (CAD) methods have been proposed for the early diagnosis of breast cancer based on histopathological images. Even though many such classification methods achieved high accuracy, many of them lack the explanation of the classification process. In this paper, we compare the performance of conventional machine learning (CML) against deep learning (DL)-based methods. We also provide a visual interpretation for the task of classifying breast cancer in histopathological images. For CML-based methods, we extract a set of handcrafted features using three feature extractors and fuse them to get image representation that would act as an input to train five classical classifiers. For DL-based methods, we adopt the transfer learning approach to the well-known VGG-19 deep learning architecture, where its pre-trained version on the large scale ImageNet, is block-wise fine-tuned on histopathological images. The evaluation of the proposed methods is carried out on the publicly available BreaKHis dataset for the magnification dependent classification of benign and malignant breast cancer and their eight sub-classes, and a further validation on KIMIA Path960, a magnification-free histopathological dataset with 20 image classes, is also performed. After providing the classification results of CML and DL methods, and to better explain the difference in the classification performance, we visualize the learned features. For the DL-based method, we intuitively visualize the areas of interest of the best fine-tuned deep neural networks using attention maps to explain the decision-making process and improve the clinical interpretability of the proposed models. The visual explanation can inherently improve the pathologist’s trust in automated DL methods as a credible and trustworthy support tool for breast cancer diagnosis. The achieved results show that DL methods outperform CML approaches where we reached an accuracy between 94.05% and 98.13% for the binary classification and between 76.77% and 88.95% for the eight-class classification, while for DL approaches, the accuracies range from 85.65% to 89.32% for the binary classification and from 63.55% to 69.69% for the eight-class classification.

## 1. Introduction

Breast cancer (BC) continues to be the most prevalent and invasive cancer in women for decades. Every year, the incidence and death rates are increasing enormously. According to the World Health Organization (WHO), the number of cancer-related deaths among women is 2.1 million per year, and in 2008, it was reported that 627,000 people died of BC [1]. Early detection is crucial for improving BC survival, based on well-known imaging modalities, such as X-ray mammography, which is considered a reference examination used by doctors to identify early breast lesions and abnormal changes in breast tissue. However, it does not allow to know whether the tumor is benign or malignant. As a result, a biopsy procedure is often carried out where the sample tissue is removed from the suspected region and sent to the laboratory for examination. The sample is studied under a microscope by a pathologist and a final report confirms or not the presence of cancerous cells in the sample. While this approach is the most reliable way to provide a diagnosis with certainty, it suffers from a subjectivity issue where a separate diagnosis can be made on the same sample and, in particular, between non-specialized pathologists. There is also the problem of workforce shortages in the field of histopathology [2], which puts the tissue sample on hold for up to two months, for example, this often occurs in Norway [3]. Thus, there is an excess demand to develop computer tools to overcome these limitations.

With the exponential growth of artificial intelligence, the computer-aided diagnosis (CAD) technique on medical imaging is increasingly becoming one of the effective ways to automatically detect and diagnose diseases at an early stage. The CAD methods of BC aim to automatically classify breast findings as benign or malignant, using intelligent approaches. With the advent of whole slide digital scanners, complete slides can now be scanned to create and produce high-resolution digital files [4,5]. This opened the door to researchers and practitioners to carry out a quantitative analysis of histopathological images through developing reliable CAD tools that facilitate the detection and diagnosis of different diseases [4,5].

Over the last few years, considerable research efforts have been made for classifying breast abnormalities based on histopathological images [6,7,8,9]. In the CAD frameworks based on conventional machine learning (CML), a set of handcrafted features of BC images are extracted and then classified using a standalone classifier. The process of classifying histopathological BC images is extremely reliant on feature extraction stage. Morphological-based features, textural-based features, fractal features, color features, and intensity-based features are the most commonly used features to differentiate between different BC image classes. However, manual feature extraction is deemed to be extremely time-consuming and often requires an extensive prior domain knowledge of the disease to define highly representative features. In addition, handcrafted features are mostly indistinguishable and suffer from what we call the *curse of dimensionality*. Such problems hampered their interpretation and the comprehension of what was learned from the input data.

In histopathology workflow, the advantages of handcrafted features CAD approaches have not been fully identified. There has been much controversy and debate about whether CAD is an efficient technique at the present level of performance [10]. The daunting nature of machine vision tasks gives rise to a key move in a new age of machine learning methods that will allow computers to learn features as data representatives at multiple levels of abstraction. These are expressed as *low-level* features such as margin and edge, *middle-level* features such as edge junctions, and *high-level* object parts [11]. The MIT technology review [12] has acknowledged the deep learning (DL) methods as one of 10 groundbreaking developments and breakthrough technologies of recent years. DL-based methods have been inherently developed to overcome the limitations of CML-based methods. Convolutional neural networks (CNNs), a class of deep neural networks, have achieved an unprecedented performance in the field of biomedical image analysis thanks to their powerful capability to automatically learn image data representation based on a fully supervised end-to-end process [13]. However, the large scarcity of very large labeled histopathological image datasets has hindered the full exploitation of CNNs, posing a major obstacle to a worthy histopathological image analysis. CNNs are characterized as data-hungry networks with a high likelihood of over-fitting in the case of limited data. Transfer learning [14,15] has been introduced as a promising alternative to the full training of CNNs, aiming to transfer the knowledge between source and target domains from similar or different applications. The standard way of performing transfer learning with deep CNNs is by using data from the target task to fine-tune a pre-trained model on the source task. Working with ImageNet [16] pre-trained models for fine-tuning remains the most common practice for many computer vision applications [17].

However, deep CNNs operate with raw input values and internal neural activations that humans find difficult to comprehend. Deep CNNs’ black-box decision-making approach is a deterrent to their use in high-risk everyday activities, where end-users are generally unfamiliar with deep learning [18]. In real-life practices, physicians rely on particular regions of an image to assess the class it belongs to and the probability of malignancy. The network performance and output, on the other hand, can only be explained in terms of its internal state (i.e., internal layer activations, values of weights, input pixels, etc.), which is far from the semantics of physicians, who are concerned with the size, shape, and other related features of the suspicious area [19]. A wide range of techniques were therefore developed to demystify deep CNNs and eliminate their *black box* bottleneck. Gradient-weighted Class Activation Mapping (Grad-CAM) [20] is a state-of-the-art heatmap visualization technique that generates a localization map using the gradients of any target class relative to the last convolutional layer. The obtained heatmap is used to visually explain where in the image the DL model is looking for making a particular prediction. Highlighting the discriminative regions in histopathological images can make pathologists entrust the network output while providing them with more clinically interpretable results.

In view of the above, this paper aims to provide an evaluative study with an explainable comparison of CML and DL approaches for the classification of histopathological BC images with respect to their magnification factors using BreaKHis dataset. First, we describe the proposed CML and DL methods which are used for classification. Second, we provide the obtained statistical results. Third, we justify the difference in classification between CML and DL methods through PCA and KPCA image features visualizations. Ultimately, we explain the decision-making process of the DL method through Grad-CAM visualizations. The main contributions of our work can be summarized as follows:

- We apply different feature extractors and classifiers to the task of histopathological BC image classification to make a full comparison. We extract handcrafted features using three feature extractor techniques (Zernike moments, Haralick, and color histogram), and the extracted feature vectors are then fused. The obtained feature vector is subsequently classified using five classical classifiers: KNN, random forest (RF), multi-layer perceptron (MLP), Adaptive Boosting (AdaBoost), and SVM.
- Based on our previous work [21] where two fine-tuned residual blocks from the ImageNet pre-trained ResNet-18 model were sufficient to achieve recent state-of-the-art results for magnification dependent classification, we are motivated to investigate the block-wise fine-tuning strategy on another deep CNN architecture, VGG-19 [22]. We extend our previous work by fine-tuning VGG-19 block-by-block aiming to figure out the best proportion of blocks to fine-tune when classifying breast histopathological images with respect to their magnification factors while examining and discussing the impact of block-wise fine-tuning on the classification results.
- Along with the comparison of classification performance, we provide a visual interpretation for the compared approaches. We use principal component analysis (PCA) and kernel-PCA (KPCA) to reduce the deep features and handcrafted features to 2D space and then visualize/compare them intuitively. Through the comparison of the visualization results, the difference in classification performance between CML and DL methods can be explained.
- We further explore the explanation of the diagnosis decision of the DL method. We obtain the activation map of the last convolutional layer from the DL model by Grad-CAM, and then visualize it using a heatmap, showing where in the input image (key areas), the DL models are looking to settle on a certain class output. Through the explanation, we can provide the diagnosis decision and the reasons for making such a decision simultaneously, which inherently helps to improve the pathologist’s trust in automated DL methods.
- To further test the adaptability and generalizability of the proposed methods, we interestingly validate them on KIMIA Path960, a more challenging and magnification-free histopathological image dataset. To our best knowledge, we are the first to provide such an explainable comparison based on different machine learning methods using two distinct histopathological datasets. The validation on the KIMIA Path960 dataset proves the good generalizability of the classification and visualization methods. Such visual validation on another histopathology dataset can further consolidate clinical trust in the proposed methods.

The remainder of this paper is organized a follows: In Section 2, we give a succinct review of related works; in Section 3, a description of our proposed methodology used in this work is provided; in Section 4, the experimental setup and the comparison and visualization results using the proposed approaches are provided and discussed; in Section 5, we conclude this work and provide some perspectives and future directions.

## 2. Related Works

In BC diagnosis, pathologists routinely inspect breast tissues visually which makes the process challenging, subjective, and time-consuming. To relieve the workload on pathologists, there has been a huge interest over the last few years for automating this process through adopting CAD systems. CAD systems based on CML methods rely essentially on extracting handcrafted features from histopathology images and use them to train specific classifiers in order to predict the final output labels. Brook et al. [6] used generic features with support vector machines to classify biopsy images into three classes using a decent data set. Belsare et al. [7] extracted gray level co-occurrence matrix (GLCM), graph run-length matrix (GRLM), and Euler number features and then classified them using linear discriminant analysis (LDA), K-nearest neighbor (KNN), and support vector machine (SVM) classifiers. The LDA achieved the best classification accuracy, which was 100% and 80% rates for the non-malignant and malignant breast histopathology images, respectively. Recently, Spanhol et al. [8] released the largest publicly available dataset (called BreaKHis) containing 7909 histopathological images and assessed the task of classifying the images into benign and malignant using six algorithms to extract the image texture features, with help of four classical classifiers. The reported accuracy results were between 80% and 85%. Sanchez-Morillo et al. [9] extracted KAZE image features along with Bag-of-Features (BOF) and fed them as inputs to a binary SVM classifier to discriminate between benign and malignant BC. The reported results were between 71.3% and 85.9%.

Due to the complexity and computational burden associated with extracting meaningful handcrafted features, the classification results reported from the above-mentioned studies were unsatisfactory and quite far from the need. To address such limitations, many researchers exploited CNNs due to their strong ability in automatic global feature learning directly form raw data images. Bardou et al. [23] proposed the classification of breast cancer into benign and malignant and into benign and malignant sub-classes using CNNs. The authors designed a CNN model and also tried an alternative approach where they fed a set of encoded features into CNN instead of the histopathological images. Bayramoglu et al. [24] also used CNNs for the classification of breast cancer into benign and malignant. The authors developed two architectures: (1) A single task CNN which is used to predict malignancy, and (2) A multitask CNN to simultaneously predict both malignancy and image magnification factor. The accuracy of the single task CNN model was 83.25% while for the multitask CNN model was 80.10%. Spanhol et al. [25] used deep network architectures which are variants of the AlexNet model [26] and LeNet to classify benign and malignant histopathological images. The authors used the BreaKHis dataset to train the CNN models where 70% of the data are used for training and 30% for testing. The highest accuracy achieved based on these models was 85%. In [27], a deep learning model with a single convolutional layer is designed with a poor accuracy of 70%. Li et al. [28] proposed a better version of the CNN model with multi-layers that allowed reaching an accuracy of 90%. In [29], Nahid et al. used a restricted Boltzmann machine along with a deep CNN, while in [30], they proposed a combination of CNN and recurrent neural networks. In [31], the same authors also built various CNN models that consider both deep learning features and handcrafted features. Baltres et al. [32] proposed a deep learning-based approach to predict an alternative recurrent score of Oncotype DX using histopathological characteristics. Zemouri et al. [33] proposed a new joint variable selection and constructive deep neural network “ConstDeepNet”-based approach. The authors applied the variable selection method to improve the training of the deep learning model. In [34], the same authors proposed another approach based on the constructive deep neural network to address the same task. Some recent studies exploited transfer learning to remedy the data scarcity while addressing this task such as in [21,35,36,37,38,39,40,41]. In [35], a combination of deep learning, transfer learning, and generative adversarial network is proposed to improve the classification performance. In [36], Song et al. represented the images by Fisher Vector encoding of the local features extracted from a pre-trained CNN model on ImageNet. The accuracies reached by the models were 87% and 90%, respectively. In [37], a similar approach was proposed with an accuracy of 90%. Double transfer learning was also tested in [38]. Boumaraf et al. [21] optimized the weights of the pre-trained ResNet-18 model [42] on BreaKHis dataset by fine-tuning the last two residual blocks to make them more task-specific. They have strengthened the performance of the proposed model by using global contrast normalization based on the target’s task image values and three-fold data augmentation. Their results outperform recent state-of-the-art methods for both magnification dependent and independent binary and eight-class classifications by a fair margin. Shallu et al. [39] fine-tuned three deep pre-trained models (VGG-16, VGG-19, and ResNet-50) to classify BC benign and malignant histopathological images. The best-reported result was 92.60% accuracy using the VGG-16 model. Saxena et al. [40] used a pre-trained CNN model as a feature extractor for the classification of breast cancer histopathological images. The process consists of dividing the images into non-overlapped segments. Then, extracting a set of features using the pre-trained CNN model. Finally, using SVM as the output classifier, Gour et al. [41] designed a model named ResHist. The model is composed of a residual learning-based 152-layered CNN.

Despite their near-human-level performance in histopathology BC diagnosis, however, DL models are frequently assumed to be a “black box” lacking sufficient interpretability to explain the human-like decision-making prediction mechanisms [43]. Hence, several methods have been recently proposed aiming to make DL models more transparent to users, to provide a deeper insight into their working strategies, and to clarify why a specific decision has been made for a particular input [44,45]. For instance, saliency maps [46], image captioning [47,48], and visual attention map [49,50] techniques are used for demystifying DL models in whole-slide histopathological image (WSI) analysis. However, we noticed that there were insufficient research studies on the visualization and interpretation of CNNs in histopathological BC diagnosis, which restricted their use in routine histopathology workflow.

## 3. Methods

Our approaches involve extracting features from histopathological images and then classifying them into their respective classes using both CML and DL approaches. The same pre-processing stage is first applied to both CML and DL methods. For the CML methods, we utilized different handcrafted feature extractors and multiple classifiers. Herein, we extract *Zernike moments* features, *Haralick* features, and *color histogram* features to represent histopathological image data, then the resulting features are fused and the obtained feature vector is further classified using KNN, RF, MLP, AdaBoost, and SVM classifiers. For the DL-based approach, we investigate the pre-trained VGG-19 model with transfer learning to automatically extract deep features and conduct classification using a softmax classifier. More details of the proposed methods are shown below.

### 3.1. Image Pre-Processing

In this stage, we pre-process the datasets by normalizing the stain-color of images and prepare them for subsequent stages.

**Figure 1:**
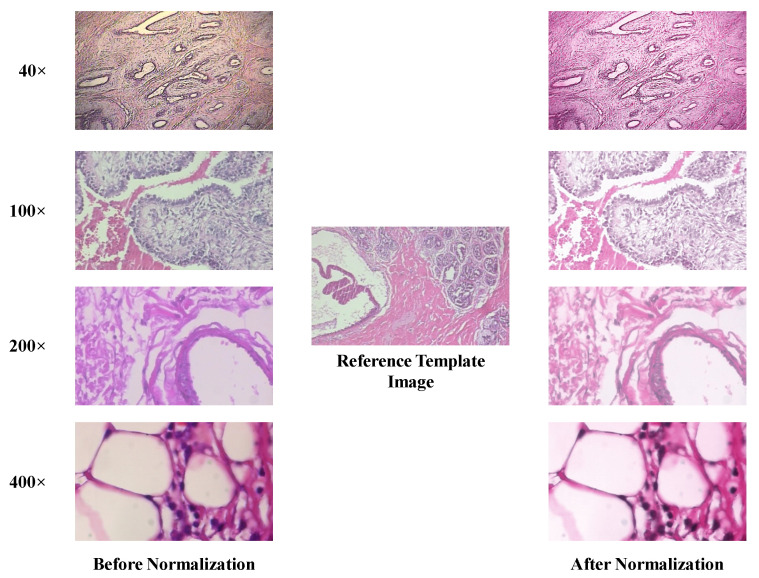
Examples of images before (**left column**) and after (**right column**) color normalization.

Histopathological images are usually acquired using different microscopes or scanners. The visualization of microscopic structures is enabled by the coloring of the tissue by chemical stains from diverse manufacturers. Consequently, a significant color variation between images can be unavoidable, which undermines the efficient development of automatic histopathological imaging techniques. To cope with this issue, many stain normalization algorithms have been introduced. In this paper, we investigate the approach proposed by Vahadane et al. [51] which changes the color of one image (source) to match that of another (target or reference) image. It integrates a regularized stain separation which is incorporated using a non-negative matrix factorization. This color normalization technique preserves the structural information of the source image after color normalization, which is very important for subsequent image analysis stages. To normalize our images, we first asked an experienced pathologist to pick a reference template image from the experimental dataset. Then we normalize the images in the dataset using the same method used in [51]. Figure 1 illustrates the effect of stain normalization on a few histopathological samples from the BreaKHis dataset. The reference template image was chosen from 40× magnification data. The first two rows are benign images in 40× and 100× magnifications, while the last two rows below show malignant images in 200× and 400× magnifications, respectively.

### 3.2. Conventional Machine Learning (CML) Approaches

**Figure 2:**
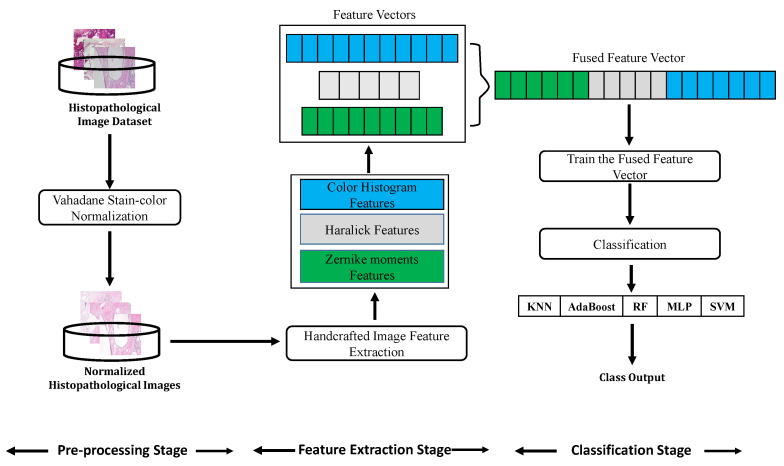
The pipeline of conventional machine learning (CML) classification methods used in our work.

The framework of CML methods for image classification usually involves different stages: image acquisition, pre-processing, feature extraction, and classification. Figure 2 shows our proposed CML pipeline used for histopathological image classification.

#### 3.2.1. Feature Extraction-Based CML Approaches

Extraction of features is a critical stage in classification pipelines. To better represent the images from different feature aspects, we exploit three different feature extractors and merge the obtained features to form a fused feature vector of 550 dimensions.

• **Zernike Moments Features:** Morphological features are essential to represent the shape and margin (smooth or irregular) of cancer cells. To quantify morphological features, we use Zernike moments [52,53] as descriptors of shape and margin which are substantially robust to noise, invariant to scale and rotation, and provide a fast and efficient computation, particularly in large image datasets. The calculation of Zernike moments from an input image includes three steps: calculation of radial polynomials, calculation of Zernike basis functions, and calculation of Zernike moments by projecting the image onto the Zernike basis functions [54]. Zernike polynomials are continuous orthogonal polynomials defined in polar coordinates over a unit disk [55]. The discrete form of the Zernike moments for an image with the size N × N is defined as follows:

(1) $$\begin{array}{cc} Z_{n,m} & =\frac{n+1}{\alpha_{N}}\sum_{c=0}^{N-1}\sum_{r=0}^{N-1}\;f\left(x,y\right)\;V_{n,m}^{*}\;\left(x,y\right)\\ \; & =\;\frac{n+1}{\alpha_{N}}\sum_{c=0}^{N-1}\sum_{r=0}^{N-1}f\left(x,y\right)R_{n,m}\;\left(p_{xy}\right)e^{-jm\theta_{cr}} \end{array}$$

where, $0\;\leq \;p_{xy}\;\leq \;1$ and $\alpha_{N}$ is a normalization factor. *n* is a non-negative integer representing the order of the radial polynomial. *m* is an integer satisfying constraints n−|m|=evenand|m|≤n representing the repetition of the azimuthal angle. $R_{n,m}$ is a radial polynomial and $V_{n,m}$ is a 2-D Zernike basis function. As a feature, Zernike moments are built using a set of polynomials and are defined inside the unit circle and the radial polynomial vector which is calculated with order *n* and repetition *m*. More details on Zernike moment computation can be found in [56]. In our work, we extract a total of 25 Zernike moments from orders 0-8 from each histopathological image.

• **Haralick Features:** Cancer cells are also characterized by their textures which reflect the pattern distribution of nuclei inside the breast tissue. To quantify the textures of histopathological images, we utilize Haralick features [57] computed from GLCM. To do so, we first convert the input color images into gray-scale. To represent different texture scales in histology images, we opt for multiple distances *d* and eight angles α, same as in [58,59]. Then, we extract the same 13 Haralick features (we only removed *maximal correlation coefficient* due to its instability). To minimize the number of derived features arising from the various values of *d* and α, we simply took the *mean* of them.

• **Color Histogram Features:** In histopathology, visualization or recognition of microscopic structures is facilitated by histological staining of cancer tissues, which is typically accomplished with Hematoxylin and Eosin. Hematoxylin colors the nuclei cells with a purple/blue color while Eosin gives pink color to other structures. Hence, the distribution of colors in an image is an important feature that needs to be quantified for the differentiation of different cancer classes. To do so, we compute the image color features based on color histogram, which represents the number of pixels in each bin that have the same color for a fixed list of color ranges. Since we have RGB histopathological images, we used histograms of 8 bins in each channel which produces a 512 feature vector. The stain-color normalization carried out at the pre-processing stage makes the color histogram features much more discriminatory due to the unified appearance of the resultant stain-normalized images.

#### 3.2.2. Classification-Based CML Approaches

In this stage, the obtained three feature vectors are first fused and the resulting fused feature vector is then trained and classified using five classical classifiers: KNN, RF, MLP, AdaBoost, and SVM. KNN is a type of learning algorithms that stores all available training data and classifies the testing data based on a similarity measure (e.g., Manhattan distance). RF is an ensemble approach for classification and regression that consists of a large number of decision trees predictors. Each decision tree in the RF outputs a class prediction and the class with the most votes becomes the model’s prediction. MLP is a special type of feed-forward artificial neural network consisting of many nodes called neurons organized in layers, namely: input layer, one or more hidden layers, and an output layer. Each layer is composed of a pre-defined set of neurons and every neuron in a layer is fully connected to the next layer. AdaBoost is an ensemble of learning approaches which uses an iterative method to learn from the errors of weak and inaccurate classifiers, and turn them into strong ones. SVM is a well-known discriminative classifier that creates a separating hyperplane in a high-dimensional space that can be used for classification and regression tasks.

### 3.3. Deep Learning (DL)-Based Approach

Recently, Deep CNNs have demonstrated an impressive performance in many computer vision applications. In our work, we exploited the transfer learning technique on one of the most state-of-the-art architectures: Visual Geometry Group Network (VGGNet) which is pre-trained on ImageNet, aiming to transfer the knowledge from natural images to BC histopathological images. VGGNet has been proposed in 2014 by Simonyan and Zisserman [22] from Oxford University. The authors investigated the assumption of getting better performance with much deeper networks. VGGNet outperforms AlexNet [26] by adding more convolutional layers and was able to get 7.3% top-5 error on 2014 ImageNet challenge. In our work, we investigated VGG-19, a successful variant of VGGNet. VGG-19 has 19 layers sequentially stacked on top of each other. It mainly consists of five VGG blocks, five max-pooling layers, and three fully-connected layers (i.e., a dense block). Each VGG block involves several standard convolutional layers having solely very small 3 × 3 filters, and each followed by a 2 × 2 max-pooling layer.

Deep learning models such as VGG-19 work best for a very large image dataset such as ImageNet, but for smaller datasets, their efficiency is typically hampered by common issues such as over-fitting. Data augmentation is the most common way to tackle the over-fitting problem during the model’s training, in which our normalized image dataset obtained after pre-processing is artificially increased in size. Here, we applied five augmentation techniques to the training input images: random horizontal flip, random vertical flip, random rotation with 90 degrees, random rotation with 180 degrees, and random rotation with 270 degrees.

Then, the augmented dataset is used to train the DL models. For training, we exploited transfer learning. We followed the same strategy proposed in our previous work [21], where we fine-tuned two residual blocks of a pre-trained ResNet-18 model to achieve recent state-of-the-art results for magnification dependent classification. Here, we extend our previous work in [21] by investigating the block-wise fine-tuning strategy on another deep CNN architecture, VGG-19 [22]. The difference here is that we tend to fine-tune VGG-19 block-by-block aiming to figure out the best proportion of blocks to fine-tune when classifying breast histopathological images with respect to their magnification factors while examining and discussing the impact of block-wise fine-tuning on the classification results. The motivation behind this sort of fine-tuning is two-fold: firstly, if we fine-tune the VGG-19 deep model (19 layers in total) with the commonly used layer-wise fine-tuning, it will require 19 per-layer fine-tuning which is a dull and time-intensive process if we consider the time required for training each fine-tuned architecture; secondly, to our best knowledge, this block-by-block fine-tuning strategy has never been tried, examined, or discussed previously in BC histopathological image classification methods, except for other imaging modalities such as brain tumor classification in MRI images [60].

**Figure 3:**
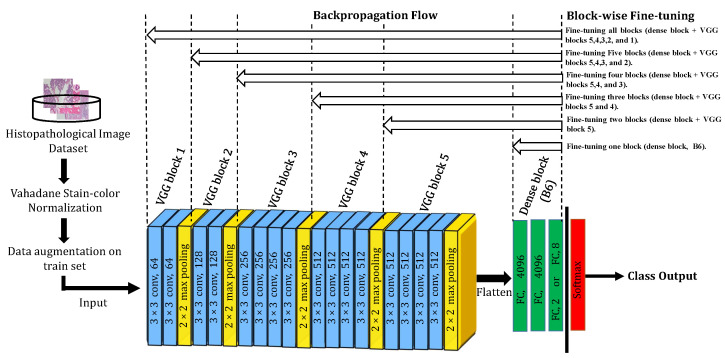
The proposed DL-based architecture and block-wise fine-tuning strategy on histopathological images.

The proposed DL approach and the followed block-wise fine-tuning strategy are illustrated in Figure 3. As can be seen, we proceed with the block-wise fine-tuning as follows: we first removed the last fully-connected layer that was originally devoted to output 1000 class categories in the ImageNet dataset and we replaced it with our customized fully-connected layer with the neuron number corresponding to the class number in our dataset. Secondly, we divided the VGG-19 architecture into six distinct blocks (B1, B2, B3, B4, B5, and B6), where the first five blocks are VGG blocks of regular 3 × 3 convolutional layers with periodic 2 × 2 max-pooling layers, and the sixth block is a dense block with three fully-connected layers. We then retrain the pre-trained deep VGG-19 model by starting fine-tuning by a block from block B6 and keeping all other VGG blocks (B1, B2, B3, B4, and B5) untrainable by freezing their learning. We progressively go deeper into the earlier blocks of the deep model, gradually resume backpropagation on them, and accordingly track the performance improvement. Finally, a softmax layer is inserted on top of the deep block-wise fine-tuned model to serve as a classifier for the desired classification tasks.

## 4. Performance Comparison and Visual Explanation

### 4.1. Setup

#### 4.1.1. Datasets

In our work, two histopathological image datasets were considered. We first used the BreaKHis dataset to build and evaluate the proposed BC classification approaches and then investigated the KIMIA Path960 dataset to validate them based on the obtained results.

**Figure 4:**
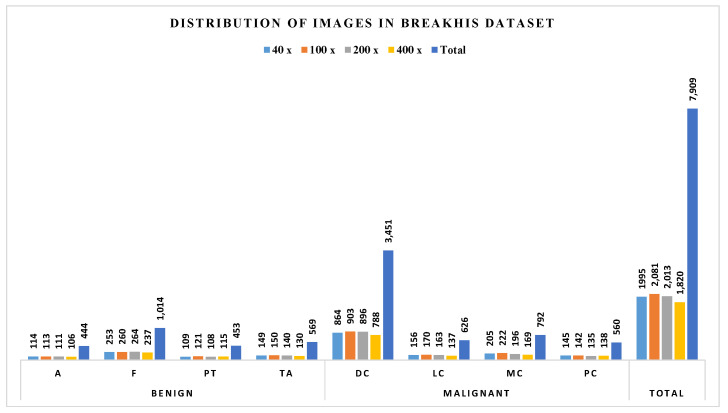
Distribution of images in the BreaKHis dataset.

BreaKHis dataset is created by Spanhol et al. [8] and is publicly available at: (https://web.inf.ufpr.br/vri/databases/breast-cancer-histopathological–database-breakhis/, accessed on 10 February 2019). It contains 7909 histopathological images acquired on 82 patients. Images in this dataset have been roughly categorized into benign BC and malignant BC. Benign BC has 4 sub-classes: adenosis (A), fibroadenoma (F), phyllodes tumor (PT), and tubular adenoma (TA). Malignant BC has 4 sub-classes: ductal carcinoma (DC), lobular carcinoma (LC), mucinous carcinoma (MC), and papillary carcinoma (PC). All the images are acquired with different magnification factors, including 40×, 100×, 200×, and 400× (see Figure 1). The distribution of images in BreaKHis is shown in Figure 4. During our analysis of BreaKHis dataset sub-classes, we noticed an image replication of the patient with ID number: 13412 in the malignant sub-classes: ductal carcinoma (DC) and lobular carcinoma (LC). This is evident and clinically intelligible because it could be a result of tumor growth inside the whole breast tissue involving different annotated lesions in the same region. However, this replication will certainly confuse the proposed CAD-based models and complicate the classification tasks. Accordingly, we have omitted the misleading images from the dataset.

**Figure 5:**
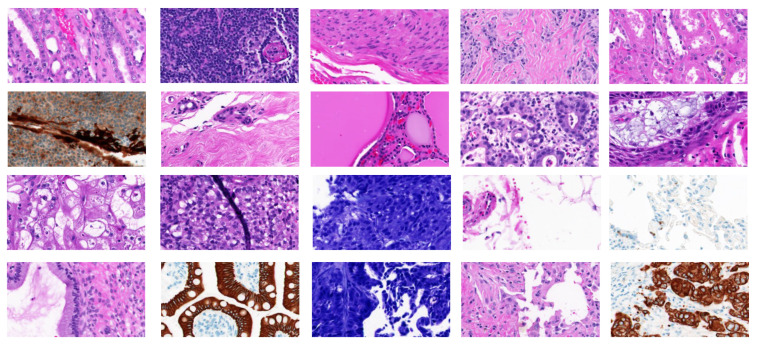
Sample images for 20 classes from the KIMIA Path960 dataset

KIMIA Path960 [61] is a collection of histopathological images from more than 400 WSI images of muscle, epithelial and connective tissue, etc. It is a magnification-free dataset, containing 960 histopathological images divided over 20 classes, which is also publicly available at: (https://kimialab.uwaterloo.ca/kimia/index.php/pathology-images-kimia-path960/, accessed on 5 April 2019). The dataset contains 20 histology scans that “visually” represent different texture/pattern types. The classes in the dataset are arranged in alphabetical letters from A to T. Forty-eight regions of interest of the same size from each WSI are down-sampled to 308 × 168 patches yielding a dataset of (20 × 48) = 960 TIF images. Figure 5 shows sample images from the KIMIA Path960 dataset.

For training CML approaches, we conduct 10-fold cross-validation where both datasets (BreaKHis and KIMIA Path960) were randomly divided into 10 subsets of the same size. Each time a subset is taken as the test set, and the remaining 9 subsets are retained as the training set, 30% of which is used as the validation set. The cross-validation process finishes when all the 10 subsets are evaluated once. The results of the 10-fold cross-validation are averaged to arrive at the final performance evaluation. For DL-based approach, we randomly split both datasets into training and testing sets. We used 70% of the data for training and we reserved the remaining 30% for testing.

#### 4.1.2. Parameter Setting

For both approaches, the images in the datasets are first resized to 224 × 224-pixel size (as required by the original VGG-19 model) before feeding them to the models. For CML approaches, we used the following parameters in all our experiments to train the five standalone classifiers: for the KNN classifier, we fixed the number of neighbors to 3 and let the other parameters in their default values. For the RF classifier, we set the number of trees to 100. For the MLP classifier, we used the default parameter values including the number of hidden layers equal to 100, the rectified linear unit (Relu) as activation function, the stochastic gradient-based optimizer (Adam) [62] as an optimizer, an L2 penalty (regularization term) parameter set to 0.0001, and an initial learning rate equal to 0.001. We also expanded the maximum number of iterations to 2000. For the AdaBoost classifier, we set the maximum number of estimators at which boosting is terminated to 50 and shrank the contribution of each classifier by a learning rate of 1. For the SVM classifier, we tested out different kernels and we kept the best parameters that provided us the highest classification results (i.e., the linear kernel with a regularization parameter C = 5).

For the DL-based approach, we optimized the weights by stochastic gradient descent (SGD) with a momentum value of 0.9. We set the learning rate to a small value of 0.001 to prevent distorting the already learned weights. To tackle over-fitting, we applied both dropout with a probability of 0.5 and weight decay regularization with a reasonable lambda value of 0.001. We set the batch size to 32 and we trained all the block-wise fine-tuned architectures for 70 epochs.

#### 4.1.3. Evaluation Criteria

To evaluate the proposed approaches, we used multiple statistical evaluation metrics, namely accuracy, recall, and precision. The accuracy measures the overall performance of the model on the testing data. The recall, also known as sensitivity, is the proportion of positives that are correctly classified. The precision, however, is the positive predictive rate.

### 4.2. Classification Performance Results

#### 4.2.1. Comparison on BreaKHis Dataset

• **Results of CML Methods**: The obtained classification accuracies using the CML approach on the BreaKHis dataset are given in Table 1.

We can see from Table 1 that: (1) different classifiers produced different accuracies for various magnification factor images; (2) binary classifications show higher accuracies than eight-class classifications; (3) for magnification dependent binary classification, the RF classifier yielded the highest classification results for 40×, 200×, and 400× data with 87.69%, 89.82%, 85.65% accuracies, respectively, except for 100× where it has been outperformed by the KNN classifier which produced 89.32% classification accuracy. SVM classifier has shown the lowest accuracy for all magnification factors except for 400× data where MLP and AdaBoost yielded the same poorest accuracy 85.31%; (4) for magnification dependent eight-class classification, the RF classifier dominates all other classifiers in all magnification factors followed by KNN, MLP, SVM, and lastly AdaBoost, respectively. Except for 400× data, where MLP showed higher classification than KNN with 62.28% accuracy.

**Figure 6:**
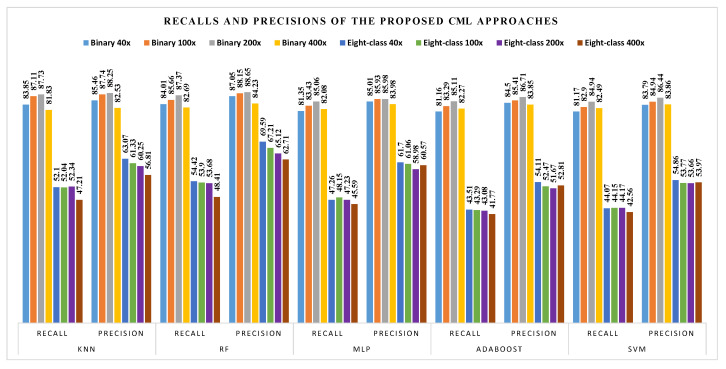
Recall and precision results (%) using the CML approaches on the BreaKHis dataset.

The corresponding recalls and precisions for different classifiers are shown in Figure 6.

• **Results of the DL Method**: The obtained classification accuracy using the DL approach with a block-wise fine-tuning strategy on the BreaKHis dataset are given in Table 2.

We can see from Table 2 that for both classification schemes, different performances are obtained with different fine-tuned blocks in the deep VGG-19 model. We can notice that the shallow block-wise fine-tuning (i.e., B6 and B6-B5) are not the optimal options for classifying breast histopathological images. The reason is that shallow block-wise fine-tuning is more beneficial in the case where the source data (ImageNet) and the target data (Histopathological images) are similar, which is not the case in our study (natural images vs medical images). Moreover, the earlier layers in CNNs learn low-level and generic image features such as color blobs and edges, while by going deeper into the network, the image features become more task-specific to the input data by learning more complex features. Hence, the best results in our study are obtained using a deep block-wise fine-tuning. Block-wise fine-tuning with B6-B3 and B6-B2 options provided us the highest results in both classification schemes. Block-wise fine-tuning with VGG-19 blocks B6-B3 is the best choice for classifying border magnification factor images (40× and 400×), while B6-B2 VGG-19 blocks are the ideal choice when conducting transfer learning for classifying in-between magnification factor images (100× and 200×), except for 100× eight-class classification, where B6-B1 yielded the highest classification result. Note that fine-tuning with B6-B1 is the optimization of all the parameters of the whole pre-trained network on the target dataset.

**Figure 7:**
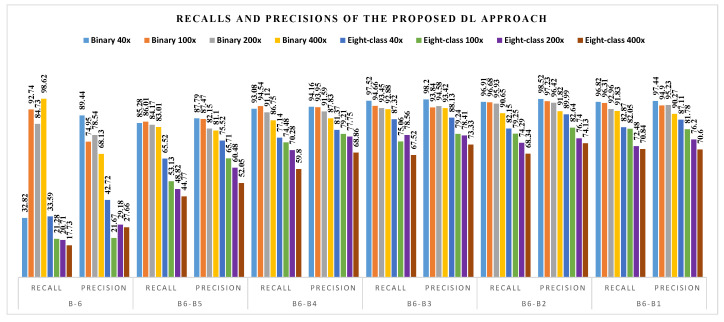
Recall and precision results (%) using the DL approach on the BreaKHis dataset.

The corresponding recalls and precisions for the different fine-tuned blocks are shown in Figure 7.

Another important thing to highlight here is that higher recall values are always preferable in the case of diagnosing BC, which has a great significance in the diagnostic histopathology practice. That is because the harm resulting from a false negative (patient remains without a diagnosis) is much more detrimental than a false positive (patient undergoes additional procedures and treatments such as chemotherapy). Hence, in case of equivalence in the accuracy results between different classifiers in CML approaches or between different blocks in block-wise fine-tuned architectures, the decision of the most appropriate classifier/architecture is based upon their corresponding recall values. The classifier/architecture with a higher recall value is selected as the most suitable one as it renders the model more sensitive to make predictions.

• **Comparison of CML and DL Methods**: From the comparison of the CML (Table 1) and DL (Table 2) methods, it can be noted that: (1) the best classification accuracies achieved using CML approaches are between 85.65% and 89.32% for binary classification and between 63.55% and 69.69% for eight-class classification. The highest obtained classification accuracies using DL approach ranges from 94.05% to 98.13% for binary classification and between 76.77% and 88.95% for eight-class classification. Obviously, the DL method achieved higher classification accuracies than the CML methods; (2) from Figure 6, we can see that the highest recall and precision values obtained using CML approaches are from binary classification of 200× data with 87.73% and 88.65% based on KNN and RF classifiers, respectively; (3) similarly, Figure 7 reveals that the best recall and precision values with DL method are from binary classification of 400× data (with B6 block fine-tuning) and 40× (with B6-B3 fine-tuning), yielding 98.62% and 98.52%, respectively.

#### 4.2.2. Validation and Comparison on the KIMIA Path960 Dataset

In this section, we further validate and compare the same CML and DL approaches on the KIMIA Path960. The obtained accuracy results for CML- and DL-based approaches are shown in Table 3 and Table 4, respectively. For CML approaches, RF classifier yielded the best classification accuracy with 93.45% followed by KNN classifier with 88.54%, while AdaBoost classifier provided 68.34% as the lowest reported accuracy. Moreover, same as in the BreaKHis dataset, the block-wise fine-tuning with B6-B3 and B6-B2 options have provided the highest results in the classification of the twenty-class KIMIA Path960 dataset with the same accuracy rate 98.26%, followed by the full fine-tuning (B6-B1) with 97.92% accuracy. Since there are two block-wise fine-tuned architectures that yielded exactly the same classification accuracy (B6-B3 and B6-B2) and based on what we have already stated, the selection of the best architecture is based on the highest recall value. Hence, B6-B2 is the best proportion to fine-tune as it has produced a 98.31% recall value. These reported results on the twenty-class KIMIA Path960 dataset validated the previous results obtained on BreaKHis dataset and prove the effectiveness and generalizability of both proposed approaches.

### 4.3. Visual Explanation

#### 4.3.1. Classification Performance Explanation

In this section, we visualize the image features utilized by both CML and DL methods, and explain the difference in classification performance between them through the visualization. For the CML method, the fused handcrafted features of 550 dimensions are collected for each image. For the DL method, we extract the deep features from the best VGG-19 models corresponding to each image data. Thus, for each image, a feature vector of 4096 dimension is derived from the last fully-connected layer of the deep fine-tuned model. Then, we use principal component analysis (PCA) and *kernel* PCA (KPCA) to reduce the dimensionality of both handcrafted and deep feature vectors and further visualize and compare them.

**Figure 8:**
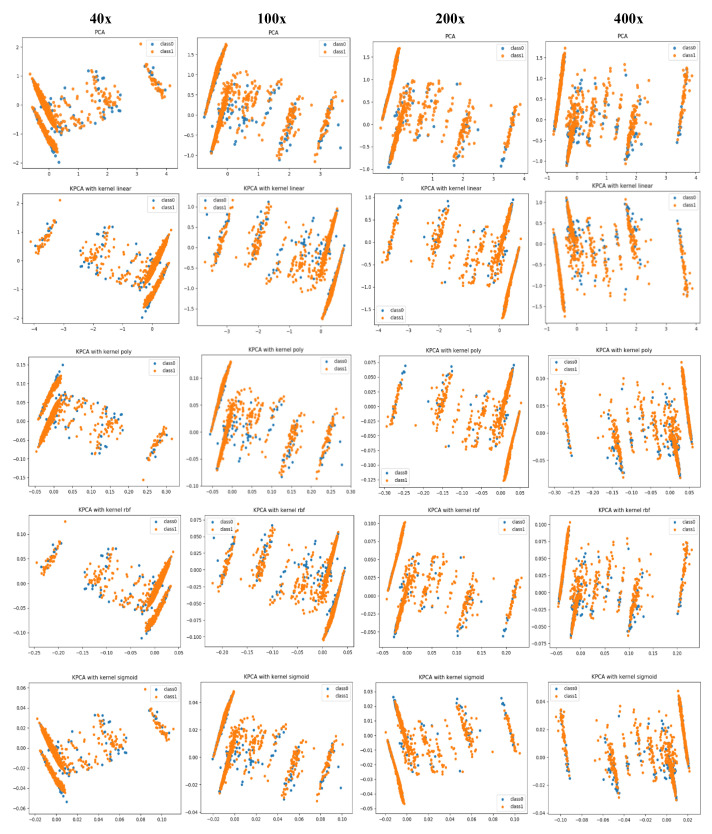
PCA and kernel-PCA (KPCA) visualizations (rows) of handcrafted features extracted using CML approaches for binary classification at each magnification factor (columns) using the BreaKHis dataset, class0 stands for benign and class1 stands for malignant.

**Figure 9:**
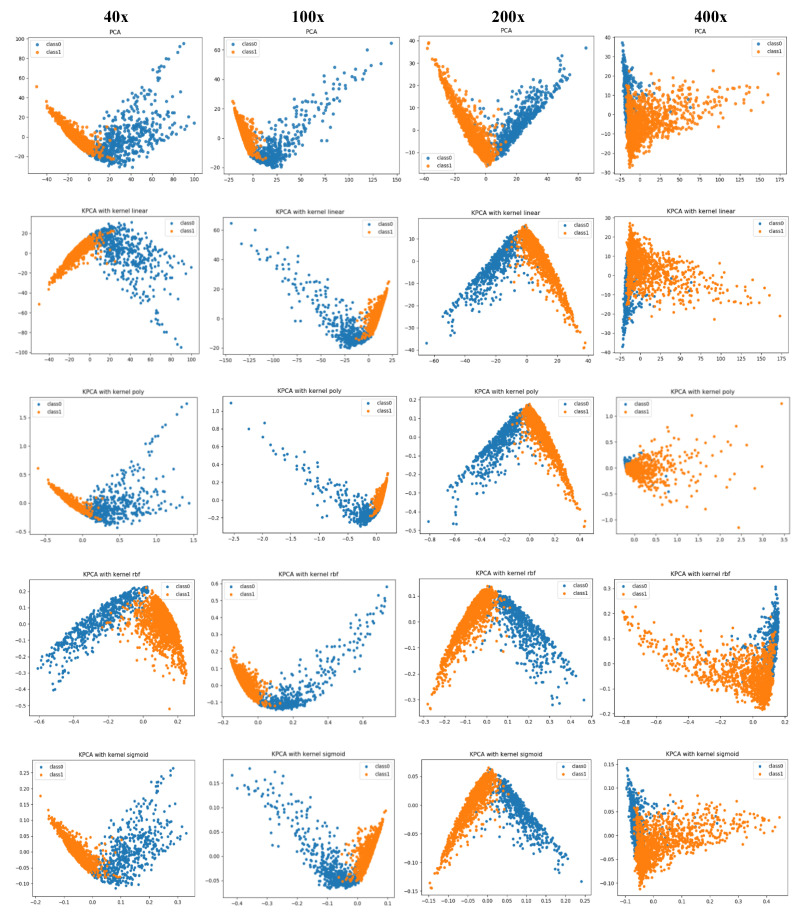
PCA and KPCA visualizations (rows) of block-wise deep fine-tuned features extracted from the best DL VGG-19 models for binary classification at each magnification factor (columns) using the BreaKHis dataset, class0 stands for benign and class1 stands for malignant.

**Figure 10:**
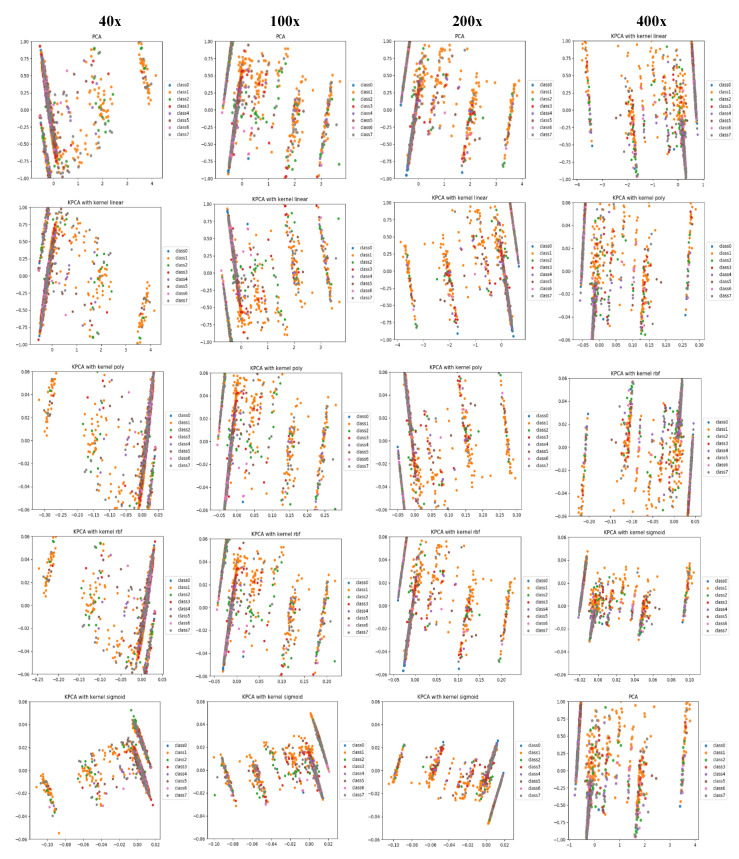
PCA and KPCA visualizations (rows) of handcrafted features extracted using CML approaches for eight-class classification at each magnification factor (columns) using the BreaKHis dataset, classes from 0 to 7 stands for A, DC, F, LC, MC, PT, TA, and PC classes, respectively.

**Figure 11:**
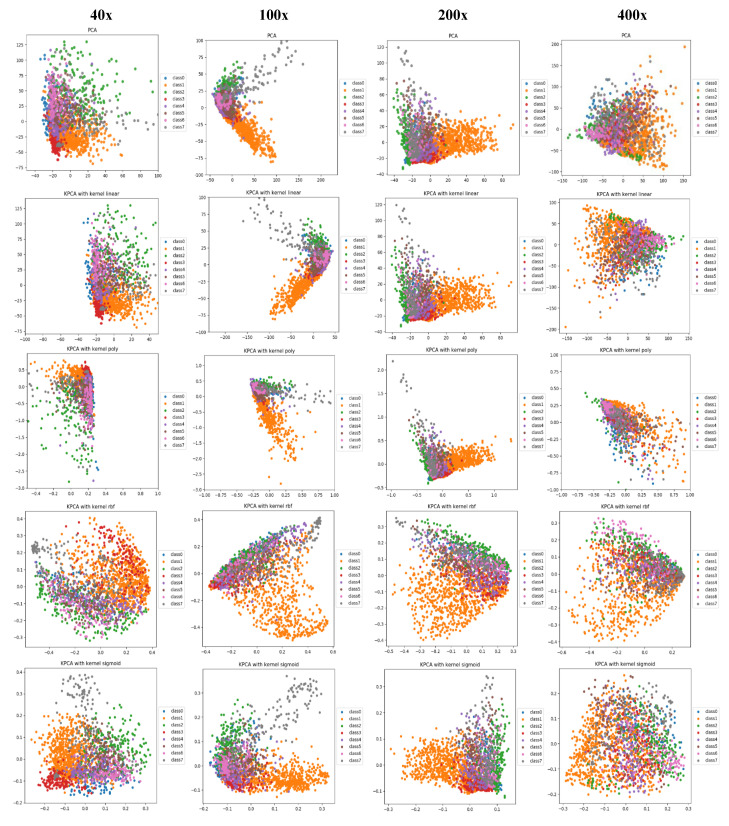
PCA and KPCA visualizations (rows) of block-wise deep fine-tuned features extracted from the best DL VGG-19 models for eight-class classification at each magnification factor (columns) using BreaKHis dataset, classes from 0 to 7 stand for A, DC, F, LC, MC, PT, TA, and PC classes, respectively.

**Figure 12:**
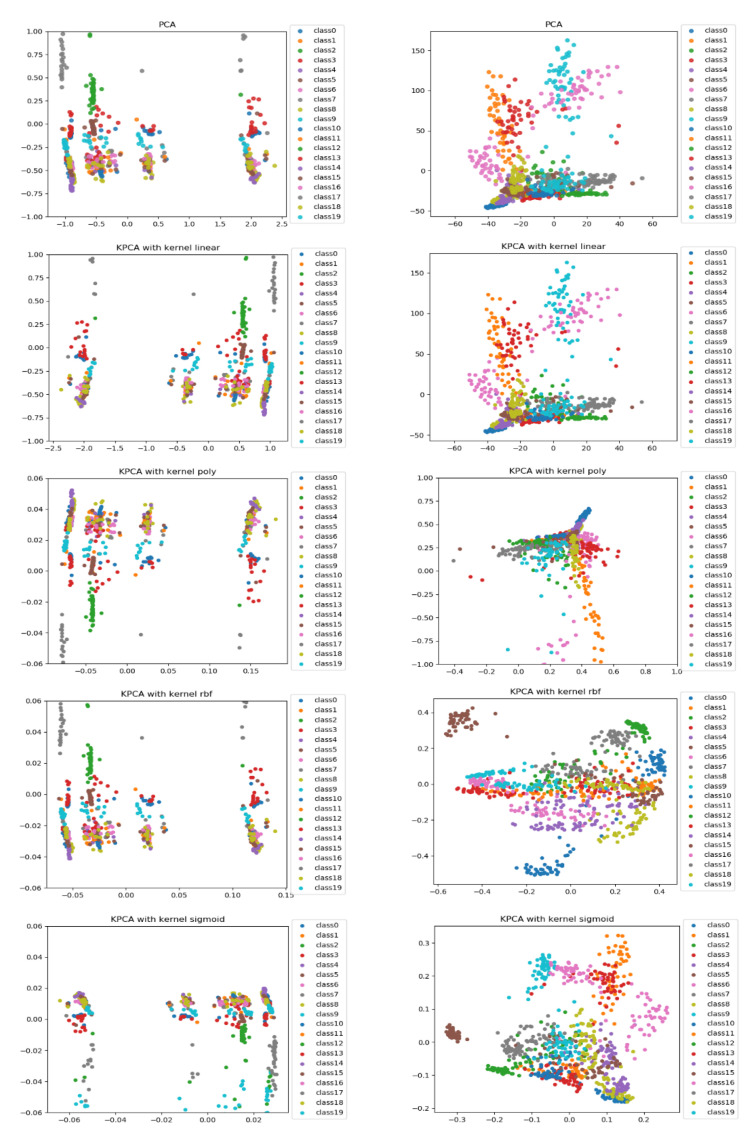
PCA and KPCA visualizations (rows) of handcrafted features extracted using CML approaches (**left**) and block-wise deep fine-tuned features extracted from the best DL VGG-19 model (**right**) for the twenty-class KIMIA Path960 classification, classes from 0 to 19 stand for A to T classes, respectively.

PCA is a linear dimensionality reduction technique that selects only the most meaningful features (in the form of components) that capture the maximum variability (i.e., statistical information) about the data. The new principal components are new variables that are linear functions of those in the original data, which successively maximize the variance and are uncorrelated with each other [63]. By this way, high-dimensional data can be projected onto a smaller 2D subspace while preserving the variance of included features as much as possible. The KPCA aims to address the drawback of the classical PCA algorithm by generalizing to a non-linear dimensionality reduction [64]. In our work, the KPCA version is used with different kernels including (*linear*, *polynomial*, *rbf*, and *sigmoid*). The PCA results of handcrafted features and deep features for magnification dependent binary classification on BreaKHis dataset are shown in Figure 8 and Figure 9, respectively. Similarly, the visualizations of features for eight-class classifications are shown in Figure 10 and Figure 11, respectively. While, the visualizations of the twenty-class KIMIA Path960 dataset are shown in Figure 12. Through the visualizations, we can notice the distribution of different classes of the reduced features. Although handcrafted features have a certain degree of separability, a huge overlap can be observed for certain classes. On the other side, the extracted deep features are much more discernible and show a promising separability between the classes (each class is grouped). Moreover, for both classification schemes of BreaKHis dataset, the 400 × data are the most complex data for classification, even with the DL approach. This has been already illustrated in the previous results of Table 2 and the PCA visualizations where there is a much significant overlap between the classes of 400× compared to other datasets. The visualization based on KIMIA Path960 also validate the effectiveness and generalizability of the proposed methods. Overall, these visualizations demonstrate that DL-based features are more potent than those of CML approaches for distinguishing breast cancer histopathological images. The visualizations of the features explain the superiority of DL method over CML methods in the classification performance described in Section 4.2.

#### 4.3.2. Classification Decision Explanation of the DL Approach

**Figure 13:**
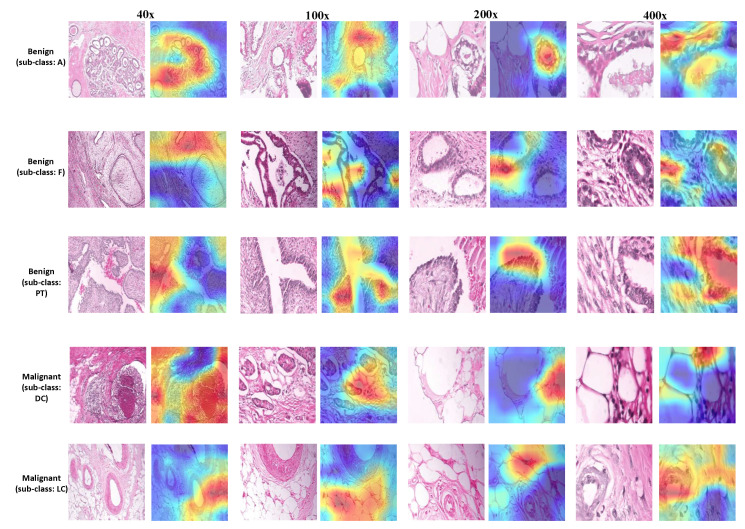
Heatmap visualizations using Gradient-weighted Class Activation Mapping (Grad-CAM) with different image samples from BreaKHis dataset. For each image row, the class and sub-class are also provided. Red regions mean high activations and blue regions mean low activations.

**Figure 14:**
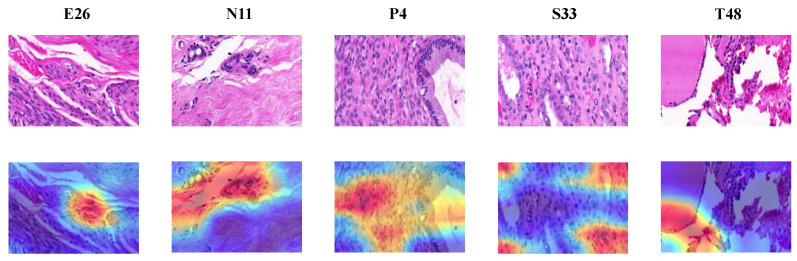
Heatmap visualizations using Grad-CAM with different image samples from the KIMIA Path960 dataset. For image column, the class is also provided. Red regions mean high activations and blue regions mean low activations.

In this section, we explore the explanation of the decision-making process of the DL method using the Gradient-weighted Class Activation Mapping (Grad-CAM) technique [20]. Grad-CAM allows to visually understand how each pixel area in the input image contributes most to the critical feature maps in the final classification output. It mainly utilizes the gradients flowing into the final convolutional layer in the network in order to produce a heatmap highlighting the key regions in the image that the model focuses for making a particular prediction. Figure 13 and Figure 14 illustrate the output obtained with different histopathological images using Grad-CAM from BreaKHis and KIMIA Path960 datasets, respectively. Note that these results are based on the best block-wise fine-tuned VGG-19 models (the ones that provided the highest performance). It can be observed from Figure 13 and Figure 14 that the morphological characteristics of the nuclei cells are the key factor influencing the output classification of the proposed models. The heatmaps generated using Grad-CAM mainly focus on the nuclei cells incorporating various and complex shape and margin features. For the BreaKHis dataset, localizing the key region in the malignant classes (e.g., DC or LC) involves looking for the nuclei cells with irregular shapes, speculated margins, and larger sizes with distinctly identifiable nucleoli and heterogeneous chromatin distribution. Compared to malignant tumors, the nuclei of benign tumors are smaller in size and tend to have round or oval shapes with well-defined or circumscribed margins. These complex patterns and structures with overlapping nuclei clusters in BC histopathological images, especially in 400× data, may sometimes mislead the DL models, resulting in a considerable number of false positive/negative outcomes that have undesirable implications for future patients’ treatment.

### 4.4. Classification Comparison with Other State-of-the-Art Methods

In this section, we compare the classification accuracy results obtained by each of our approaches with similar state-of-the-art methods. Note that our main purpose in this paper was not to achieve the current state-of-the-art performance results but to prove the applicability of various machine learning techniques for BC diagnosis and analyze their efficiencies while providing a visual explanation/interpretability of classification/decision-making in a comparative and conclusive manner. Nevertheless, our results still show a superiority over similar methods in the literature for the magnification dependent classification of BreaKHis dataset (Table 5 and Table 6) as well as for the twenty-class classification of the KIMIA Path960 dataset (Table 7). Note that for binary classification of the BreaKHis dataset (Table 5), our proposed CML approach outperforms those of Spanhol et al. [8] and Sanchez-Morillo et al. [9], and our DL-based approach outperforms the deep learning architectures proposed by Bayramoglu et al. [24] and Spanhol et al. [25]. Similarly, For eight-class classification (Table 6), both of our CML and DL-based approaches outperform different counterparts introduced by Bardou et al. [23]. For the twenty-class KIMIA Path960 classification (Table 7), our proposed CML approach outperforms the CML approach proposed by Kumar et al. [61], while our proposed DL approach shows superiority over those proposed by Kumar et al. [61] and Dif and Elberrichi [65].

## 5. Conclusions

In this paper, we have provided a comparative study with visual explanation of conventional machine learning and deep learning approaches for magnification dependent histopathological breast cancer image classification. The DL-based approach based on transfer learning with *deep* block-wise fine-tuning shows a remarkable performance superiority compared to CML approaches. The highest classification results achieved using CML approaches were between 85.65% and 89.32% for binary classification and between 63.55% and 69.69% for eight-class classification, while the best obtained classification accuracies using the DL approach range from 94.05% to 98.13% for binary classification and between 76.77% and 88.95% for eight-class classification. The PCA and KPCA visualization results of handcrafted and deep features explained and demonstrated the superiority of DL-based methods in classification accuracy. Furthermore, the visualized attention maps with Grad-CAM provided supporting regions for the diagnostic decisions and explained the decision-making process of DL-based methods. It has been shown that morphological features of nuclei cells are the main factor influencing the DL model’s predictions in BC histopathological images. We hope that our results will be clinically useful for the development of innovative diagnostic histopathology approaches not only for the diagnosis of BC, but also for different forms of tumors in different organs of the human body.

In the future work, we will investigate much deeper pre-trained architecture such as GoogleNet, ResNet, DenseNet, Inception V3 along with the block-wise fine-tuning strategy to demonstrate the obtained results of the current study and build more robust classifiers with better generalization abilities. Future investigations will be also on clinically interpreting the model’s misclassifications aiming at demystifying deep CNNs and eliminating the black box bottleneck of deep learning in BC histopathology.

## Figures and Tables

**Table 1:** The classification accuracy results (%) using the CML approaches on the BreaKHis dataset.

Classification Scheme	Magnification Factor	KNN	RF	MLP	AdaBoost	SVM
Binary	40×	87.04	87.69	85.85	85.67	85.26
	100×	89.32	88.94	87.18	86.86	86.51
	200×	89.75	89.82	88.26	88.16	87.97
	400×	84.53	85.65	85.31	85.31	85.38
Eight-class	40×	65.28	68.44	64.41	56.98	58.16
	100×	65.59	68.17	65.08	56.90	58.37
	200×	67.02	69.69	66.42	59.02	60.44
	400×	60.40	63.55	62.28	55.10	56.46

**Table 2:** The classification accuracy results (%) using the DL approach on the BreaKHis dataset (Bi means the fine-tuned blocks).

Classification Scheme	Magnification Factor	Accuracy Based on Block-Wise Fine-Tuning Strategy					
		B6	B6-B5	B6-B4	B6-B3	B6-B2	B6-B1
Binary	40×	53.23	88.27	94.56	98.13	97.96	97.45
	100×	74.27	88.76	94.79	94.95	97.39	96.09
	200×	73.74	85.52	93.10	94.95	96.63	94.95
	400×	68.03	83.27	89.03	94.05	92.38	91.64
Eight-class	40×	31.80	74.83	80.95	88.95	88.27	85.88
	100×	44.79	65.96	79.15	79.80	83.22	83.71
	200×	49.49	60.44	76.94	79.63	80.64	79.12
	400×	47.03	56.69	67.29	76.77	72.49	76.39

**Table 3:** The classification accuracy results (%) using the CML approaches on the KIMIA Path960 dataset.

Classification Algorithm	Accuracy	Recall	Precision
KNN	88.54	87.25	88.49
RF	93.45	92.89	93.47
MLP	83.19	82.64	80.50
AdaBoost	68.34	68.02	64.77
SVM	74.37	74.19	71.62

**Table 4:** The classification accuracy results (%) using the DL approach on the KIMIA Path960 dataset (Bi means the fine-tuned blocks).

Block-Wise Fine-Tuning Strategy	Accuracy	Recall	Precision
B6	68.06	68.00	74.51
B6-B5	89.58	90.13	91.17
B6-B4	96.53	96.49	96.37
B6-B3	98.26	97.96	98.12
B6-B2	98.26	98.31	98.48
B6-B1	97.92	98.02	98.23

**Table 5:** Comparison with state-of-the-art methods for benign and malignant classification of BreaKHis dataset.

Reference	Method Used	Accuracy Performance (%)			
		40×	100×	200×	400×
Spanhol et al [8].	Completed LBP features with SVM classifier.	77.40	76.40	70.20	72.80
Sanchez-Morillo et al [9].	KAZE features combined with Bag-of-Features with binary SVM classifier.	85.90	80.40	78.10	71.30
Bayramoglu et al [24].	A deep CNN model which is variant of AlexNet	89.60	85.00	84.00	80.80
Spanhol et al [25].	Single task architecture based on a deep CNN model with a softmax layer on top.	83.00	83.10	84.60	82.10
**This work**	Zernike moments, Haralick, and color histogram features all fused and classified with five standalone classifiers.	**87.69**	**89.32**	**89.82**	**85.65**
	Block-wise fine-tuned VGG-19 model with softmax classifier on top.	**98.13**	**97.39**	**96.63**	**94.05**

**Table 6:** Comparison with state-of-the-art methods for eight-class classification of BreaKHis dataset.

Reference	Method Used	Accuracy Performance (%)			
		40×	100×	200×	400×
Bardou et al [23].	SURF features encoded with bag of words (BoW) and classified with SVM.	49.65	47.00	38.84	29.50
	SURF features encoded with locality constrained linear coding and classified with SVM.	55.80	54.24	40.83	37.20
	Deep CNN features (trained from scratch) with KNN classifier on top.	70.48	68.00	70.08	66.38
	Deep CNN features (trained from scratch) with Linear SVM classifier on top.	72.35	67.68	66.45	64.95
**This work**	Zernike moments, Haralick, and color histogram features all fused and classified with five standalone classifiers.	**87.69**	**89.32**	**89.82**	**85.65**
	Block-wise fine-tuned VGG-19 model with softmax classifier on top.	**98.13**	**97.39**	**96.63**	**94.05**

**Table 7:** Comparison with state-of-the-art methods for twenty-class classification of KIMIA Path960 dataset.

Reference	Method Used	Accuracy Performance (%)
Kumar et al [61].	LBP features with two types of distance measures (Chi-squared and Euclidean distance).	90.62
	Bag of visual words (BoVW) features with histogram intersection kernel SVM classifier.	96.50
	Deep features based on pre-trained AlexNet and VGG-16 models.	94.72
Dif and Elberrichi [65].	Transfer learning based on Inception-v3 CNN architecture, six histopathological source datasets, and four target datasets.	98.18
**This work**	Zernike moments, Haralick, and color histogram features all fused and classified with five standalone classifiers.	93.45
	Block-wise fine-tuned VGG-19 model with softmax classifier on top.	**98.26**

## Data Availability

This study has been conducted using two publicly available datasets namely, BreaKHis and KIMIA Path960, the links for downloading these datasets are provided within the manuscript.
